# The Medical Officer's Library

**Published:** 1914-04-25

**Authors:** 


### THE MEDICAL OFFICER'S LIBRARY.

### The Barometer of Medical Progress.

The Medical Annual. Thirty-second Year. (Bristol: J. Wright and Sons. 1914. Pp. 800. Price 8s. 6d. net.)

A year of great activity in all kinds of therapeutic innovations has entailed a corresponding increase in size of that barometer of medical progress, the *Medical Annual*. In confessing to an additional hundred pages over last year, the editor adds the pious hope that he will be able to lop them off again in next year's issue. We doubt it; and, familiar as we are with the exceeding merit of brevity in the digests which make up a large part of the *Annual*, we are not sure that we share his hope. For the *Medical Annual* is so amazingly efficient, interesting, and practical that it is difficult to spare any of its numerous pages. As in previous years, there are some authoritative reviews of particular branches of therapeutics, prefixed to the usual alphabetical dictionary of all that is new in medicine and surgery. This year the subjects chosen for special consideration in this way are radio-activity and electro-therapeutics, thorium, and tuberculin therapy. Pellagra receives full description by an expert writer, with some good coloured photographs of the skin lesions; this is merely one instance out of many which could be quoted to show what a mine of information the volume is on recondite as well as upon common topics. In all its now numerous sections and branches of information the *Medical Annual* maintains to the full its splendid reputation of the past.

### A New Text-book on Surgery.

The Practice of Surgery. By Russell Howard, M.S., F.R.C.S. (London: Edward Arnold. 1914. Pp. 1,227. Price 21s. net.)

It is some time since a new text-book on surgery has appeared of about the size and scope of this one, though some of the older books have been kept well up to date in successive editions. The whole tone of Mr. Howard's teaching is practical and sensible; and his book is certain of wide popularity among medical students, for it contains just what they want for examinations of the standard of the ordinary "Conjoint." That is to say, ætiology and diagnosis are dealt with a good deal more fully than treatment, which is distinctly sketchy.

We have formed a high opinion of this text-book as a student's manual. It is clear and concise, as well as reliable and accurate. Those specialties in which great progress has been made of late years—for instance, orthopædics—receive correspondingly increased attention and space, compared with that which would have been allotted to them a few years ago; though even then limitations of space have obliged the author to omit much that he would doubtless have wished to include. Thus, directions for the use of special instruments are very much curtailed. Sigmoidoscopy is dismissed in a dozen lines, and cystoscopy is passed over altogether. Details of operative technique are wisely omitted, as it is impossible to include them satisfactorily in a general text-book of surgery on these lines. The same applies to ophthalmic and gynæcological surgery, which are also not touched upon; and in our opinion the author would have been well advised to omit also the chapters on diseases of the throat, nose, and ear.

We are well aware that many exaggerated ideas are prevalent as to the powers of radium, more especially in the treatment of malignant diseases. But even then the budding doctor ought to know something of what radium will not do, and also of what it sometimes will do. Mr. Howard to all intents and purposes omits radium from his teaching altogether; from which it may be inferred that he thinks very little of it. Medical students, however, and medical men ought not to be left to draw inferences when they study text-books, but to receive some authoritative teaching. A short section on the lines of the article in our Special Number (The Hospital, March 21, 1914) would have adequately met the case, and we hope that in preparing future editions this point will be carefully considered.

In nearly every other respect the new text-book is admirable. Nor must we omit to praise the type, get-up, and illustrations of the book, which reflect the greatest credit on the publisher as well as upon the author. We prophesy for it a speedy and lasting popularity.

### Still upon Goodhart.

The Diseases of Children. By Sir James F. Goodhart, Bart., M.D., F.R.C.P. Tenth Edition. Illustrated. Edited and Revised by G. F. Still, M.A., M.D., F.R.C.P. (London: J. and A. Churchill. 1913. Price 16s. net.)

It is a long cry back to 1885, when the first edition of Goodhart's text-book was published; and, in spite of the changes that have taken place since then in medical teaching, it cannot be but flattering to the author to realise that no inconsiderable proportion of his work still remains a sound and authoritative exposition. The text-book itself has, naturally, been subjected to many revisions in the course of ten editions, and has inevitably grown in bulk; but, fortunately, no attempt has been made to eliminate the personal pronoun which is really the soul of the book. As Dr. Still justly points out, "this book still owes, as it has always owed, its charm and value not only to the personal experience, but also to the personality of the original author." As a manual of instruction and of real assistance to the practitioner, we imagine this book can hardly be denied first place among English text-books on the diseases of children. The authors have not been too ready in giving space to every fresh so-called advance in pathology or treatment, but have been content with expounding their well-tried experience. This is, indeed, one of the features which has made the book so successful in the past, and will continue to make it acceptable for many years to come. One criticism must, however, be made concerning the illustrations. In number, these are far too few for such a book, and even so, one of them could well be spared; the so-called skiagram reproduced on page 443 is truly an extraordinary affair, and we are at a loss to understand how such a travesty of radiography could have been allowed to remain when instructive plates are not difficult to come by.

The subject of the specific infectious fevers always forms a part of text-books on the diseases of children, and as these diseases can but rarely be described adequately by the authors, this section is usually a compilation, whereas it should be entrusted to some fever hospital expert who can write first hand. In the book under notice it is a matter for regret that such a course was not adopted.

Among the wholly excellent and practical suggestions for the treatment of rheumatism in children, it is somewhat surprising to see included with the tonics the unusual adjunct of stout!

### The Pocket Guide Series.

Wounds and their Management. By J. K. Watson, M.D.

Medical and Surgical Dressings. By M. R. Hosking. (The Scientific Press. 1914. Price 1s. each net.)

These two latest additions to the Pocket Guide Series well maintain the practical character and high standard of the volumes already issued. Dr. Watson is, of course, an expert of proved eminence as an author of text-books for nurses, and his contribution to the Pocket Guide Series displays those qualities of orderly exposition and sound teaching which have made his previous writings so popular. Sister Hosking's book on dressings is quite as useful in its own sphere, and is just the sort of practical introduction to the subject which the junior nurse or the newly appointed dresser will find of the greatest use. The only slip we have detected is in Fig. 28, which surely ought to show the constricting bandage round the arm above, instead of below, the point of entrance of the needle into the vein.

The Biology of the Blood Cells. By O. C. Gruner, M.D. (Bristol: J. Wright and Sons. Pp. 392. Price 21s. net.)

In his preface Dr. Gruner modestly claims that he supplies only an introduction to the study of the subject of hæmatology and refers to the larger text-books. Yet twenty-five years ago, we suppose, everything known about the blood cells could have been packed into a volume a tenth of the size of this one! It is true, no doubt, that a professional hæmatologist would not regard this book as encyclopædic, but all the same it contains a huge store of information, displayed to the best advantage by author and publisher, and well illustrated. We regard this as one of the best text-books on the subject, midway between those which are elementary and those which are so advanced as to be of value only to research students.

